# Effects of AIDiet intervention to improve diet quality, immuno-metabolic health in normal and overweight PCOS girls: a pilot study

**DOI:** 10.1038/s41598-024-54100-1

**Published:** 2024-02-12

**Authors:** Małgorzata Mizgier, Barbara Więckowska, Dorota Formanowicz, Giovanni Lombardi, Alicja Brożek, Marcin Nowicki, Krzysztof Durkalec-Michalski, Witold Kędzia, Grażyna Jarząbek-Bielecka

**Affiliations:** 1 Department of Sports Dietetics, Chair of Dietetics, Faculty of Health Sciences, Poznan University of Physical Education, Królowej Jadwigi 27/39, 61-871 Poznan, Poland; 2 Department of Computer Science and Statistics, Poznan University of Medical Sciences, 60-806 Poznan, Poland; 3 Chair and Department of Medical Chemistry and Laboratory Medicine, Poznan University of Medical Sciences, 60-806 Poznan, Poland; 4 Laboratory of Experimental Biochemistry and Molecular Biology, IRCCS Istituto Ortopedico Galeazzi, 20157 Milan, Italy; 5 Department of Athletics, Strength and Conditioning, Poznań University of Physical Education, 61-871 Poznan, Poland; 6 Division of Developmental Gynaecology and Sexology, Department of Gynaecology, Poznan University of Medical Sciences, 60-535 Poznan, Poland

**Keywords:** Polycystic ovary syndrome, Adolescents, Obesity, Inflammation, Mediterranean diet, Endocrine reproductive disorders, Nutrition

## Abstract

This study was conducted in two groups of girls with PCOS (polycystic ovary syndrome) categorized as slim (group N) and overweight-to-obese (group Ov/Ob). The study's primary outcome was to assess the impact of a 12-week anti-inflammatory diet (AIDiet) intervention, without energy deficit, on daily diet quality improvement, evaluated according to the KIDMED index. The secondary outcome was improving inflammatory, redox, hormonal, and metabolic statuses. In the study, which was completed by 13 girls from the Ov/Ob group and 19 girls from the N group, a significant improvement in the mean KIDMED score was obtained. Moreover, the intervention significantly improves concentration of total antioxidant capacity (TAC), fasting insulin, and the homeostatic model assessment for insulin resistance (HOMA-IR) index, in the Ov/Ob group, while both groups experienced a reduction in the concentration of interleukin (IL)-1 and IL-6, tumour necrosis factor (TNF-α), and androstenedione. The AIDiet intervention effectively improved the quality of the subjects' diets, which was associated with the improvement of hormonal and immuno-metabolic markers. However, these changes in normal-weight patients were observed regardless of body weight reduction. ClinicalTrials.gov Identifier NCT04738409.

## Introduction

Polycystic ovary syndrome (PCOS) is one of the most common hyperandrogenic syndromes. Hyperandrogenism in women is a state of increased production of androgens hesitating into menstrual disorders, anovulation, hirsutism, and more commonly seborrhoea and acne. In women with hyperandrogenism, metabolic dysfunctions, excessive body weight, increased adiposity, atherosclerosis, diabetes, and insulin resistance are significantly more common, and infertility is diagnosed more often^1–4^

PCOS is also characterized by chronic, low-grade inflammation and a deregulated redox balance that leads to oxidative stress (OS)^5–8^. On the other hand, a low-grade inflammatory state may contribute to the development of metabolic complications observed in adult and adolescent PCOS patients^9–12^.

Lifestyle interventions, e.g., diet and physical exercise, are recommended as both preventive treatment against complications and as a therapeutic strategy to treat PCOS in women in order to improve metabolic and hormonal profiles^13^. However, there is insufficient evidence to argue that any particular type of energy-balanced diet has a better effectiveness than other types of diet^13^. It is proven that the therapeutic effectiveness of a dietary intervention results needs at least 5–10% reduction in body weight^14,15^. To achieve weight loss in overweight women, the recommendation is to implement an energy deficit with a 30% reduction in daily caloric intake, i.e., a 500–750 kcal reduction (1200–1500 kcal/day)^13^. However, it should be emphasized that PCOS also occurs in slim girls, who, however, exhibit common features and metabolic dysfunctions typical of those who are overweight and obese. In these cases, the implementation of reduction diets is unnecessary^16^. Therefore, effective therapeutic strategies should be defined. These interventions should not be necessarily related to energy restriction only; on the contrary, various dietary models, the composition and type of diet, and the phenotype of patients (slim/obese), should be all taken into account. The final aim should not be only weight loss, where needed, but also the effective improvement of the manifestations typical of PCOS^17^. This is the most important aspect given that we previously demonstrated that nutritional habits in PCOS girls differ unfavourably from those of their healthy peers, with a higher content of fats, cholesterol, and sugars and a lower content of fibres^12^.

An internationally recognized model of diet associated with beneficial eating patterns is the Mediterranean Diet (MD). Adhering to the MD means eating foods rich in fibres and vegetal proteins, among other things. This diet regimen is rich in vegetables and fruits, nuts, wholegrain cereals, and fish and contains compounds with antioxidant and anti-inflammatory properties^18–23^. It has been proven that regular consumption of MD is associated with beneficial changes in the concentration of inflammatory markers such as C-reactive protein (CRP), interleukin (IL)-6, and tumour necrosis factor (TNF)-α, and favourable changes in clinical and metabolic parameters, including weight, body mass index (BMI), waist circumference (WC), blood glucose, and insulin^24^. So far, the extent to which a dietary intervention based on a diet with increased content of natural antioxidants and anti-inflammatory nutrients can improve the biochemical and clinical characteristics of adolescent girls with hyperandrogenism and PCOS, normal weight, overweight, and obese has not been assessed.

The results obtained in our previous studies were the premise for testing the MD diet's effect on inflammation, oxidative stress (OS), and metabolic health in girls with PCOS. We observed that in adolescents with PCOS, grouped according to the body weight phenotype, there were inverse correlations between the concentration of inflammatory markers and redox balance and the supply of proteins, including plant proteins, carbohydrates, and fibres, and a positive correlation between the consumption of cholesterol and the inflammatory marker C-Reactive Protein (CRP)^8^. We also proved that increasing the supply of vegetable protein by 10 g per day significantly reduced the probability (OR, odds ratio) of overweight and obesity in girls with PCOS. The opposite relationship was observed in the case of consuming products with a medium and high glycaemic index (GI), i.e., products usually characterized by a lower fibre content^4^. These findings needed to be confirmed by further randomized trials to identify and implement a model of nutrition in girls with PCOS. Based on this background, this study aimed at verifying the effectiveness of a 12-week AIDiet intervention, based on the principles of MD, but without the implementation of energy restrictions, in girls with PCOS with normal (group N) and excessive body weight (group Ov/Ob).

This pilot study is based on the hypothesis that the implementation of a 12-week AIDiet dietary intervention will improve the perception of the diet quality in girls with PCOS. In addition, the effects on metabolic-inflammatory parameters will be investigated and, namely, on glycemia, insulinemia, blood levels of inflammatory markers (CRP, TNF-α, IL-1, IL-6), oxidoreductive (malondialdehyde-MDA, TAC) and hormonal (androstenedione, dehydroepiandrosterone sulphate-DHEA-S), both in overweight and obese girls with PCOS and those with normal weight.

To the best of our knowledge, this is the first study that proposes a dietary intervention using an anti-inflammatory diet based on MD principles in girls with PCOS, not only overweight and obese but also slim adolescent females. The results obtained will allow us to assess whether and to what extent this intervention has potential as a non-pharmacological therapy in adolescent women with PCOS, regardless of implementing an energy deficit.

## Materials and methods

### Study design and participants

This trial continues the HAStudy project conducted in 2018–2020, retrospectively registered 04/02/2021, ClinicalTrials.gov Identifier: NCT04738409. The study involved patients of the Department of Gynaecology and Perinatology, Gynaecology and Obstetrics Hospital of the Poznan University of Medical Sciences, Poland. Participation in the research was voluntary. The participants were not paid for their participation, but the participants who finished the study and their parents were provided with the test results. The enrolled girls were all Caucasian, and Polish-speaking. Age, body mass, height, and BMI were (1) in the Ov/Ob group: 15.08 ± 1.55 yrs, 83.82 ± 11.59 kg, 1.66 ± 0.06 m, and 30.13 ± 4.11 m/kg^2^; and (2) in the N group: 16.37 ± 0,96 yrs, 58.11 ± 6.83 kg, 1.66 ± 0.07 m, and 20.86 ± 2.24 m/kg^2^, respectively. They were diagnosed with PCOS according to the ESHRE/ ASRM Rotterdam 2003 PCOS criteria^1^. All the female patients with PCOS who participated in the study had irregular menses and the classic subtype with oligomenorrhea and hyperandrogenism. The characteristics of the subjects and the requirements for exclusion and inclusion in the study were described in detail previously^8^.

59 patients with PCOS were qualified for the study, including 37 slim girls, and 22 being overweight and obese. Diagnosis and classification of overweight and obesity were based on BMI, according to the World Health Organisation (WHO) for children aged 5–19 years^25^. From each weight category, 20 subjects were selected and they were assigned to one of the two groups: Ov/Ob (overweight and obese group, n = 20) and N (non-overweight and non-obese group, n = 20). Both groups underwent a 12-week dietary intervention.

Study participants were not blinded, as both groups received the same dietary intervention. Only responsible for biochemical analyses laboratory personnel were blinded.

### Description of the intervention

**Figure 1:**
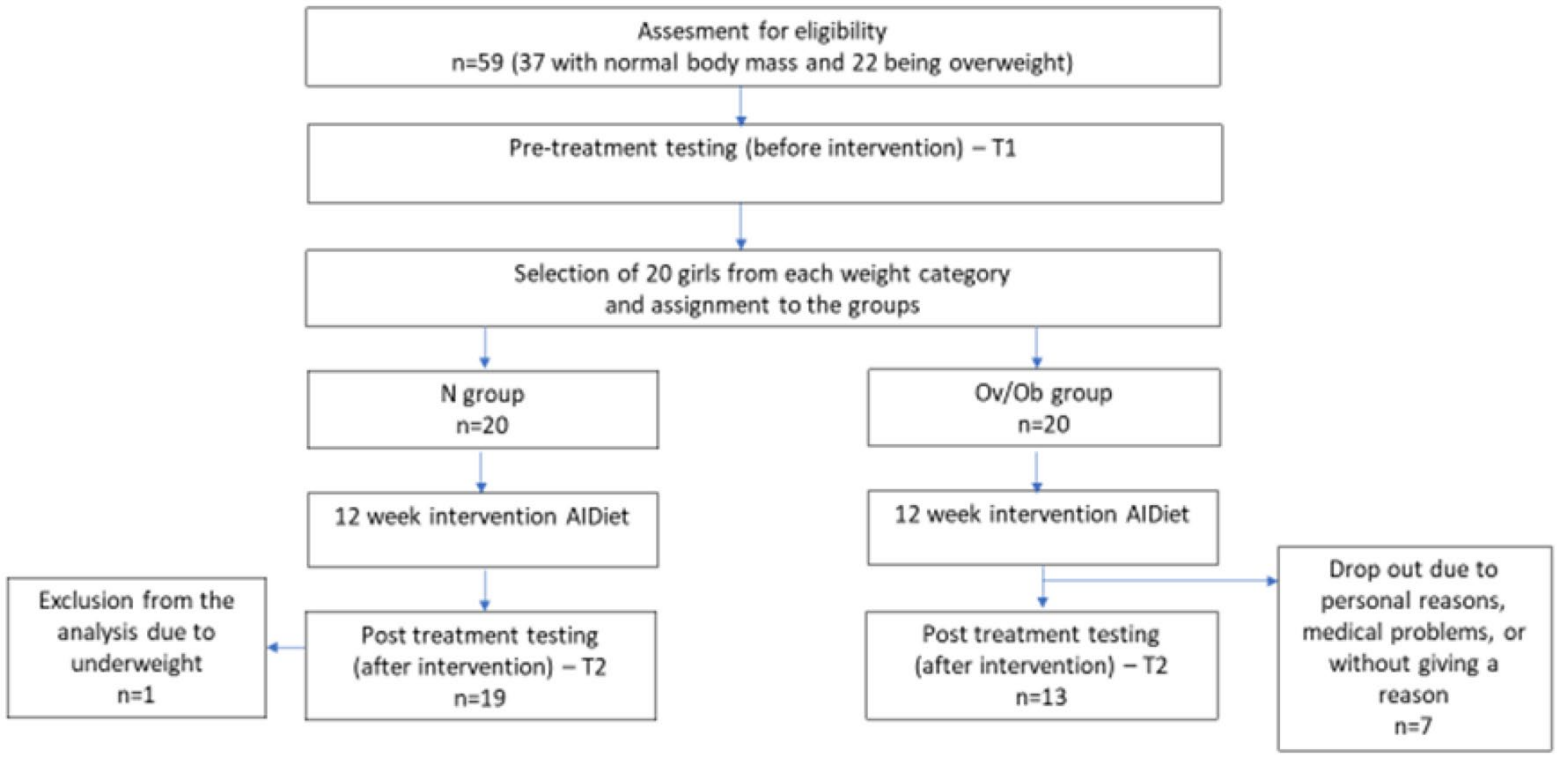
Flow chart of the study design. N group—a group of slim PCOS girls being on the anti-inflammatory diet (AIDiet). Ov/Ob group—a group of PCOS overweight/obese girls being on the anti-inflammatory diet (AIDiet).

As part of the dietary intervention, the study participants were asked to visit the study site, i.e., at the beginning of the study (T1) and after 12 interventions (T2). The course of the study is presented in Fig. 1.

After initial medical, biochemical, anthropometric examinations, and evaluation of the diet (T1), patients were assigned to the following groups: Ov/Ob and N. Then, both groups underwent the same 12-week AIDiet intervention without imposing energy restrictions. Before implementing the diet, all young women underwent an education process: during a visit to a gynaecologist who works in gynaecology of developmental age and a certified dietician.

During an individual visit to a gynaecologist (G.J-B), girls were made aware of the risks associated with PCOS and possible therapeutic effects due to lifestyle changes. One hour-and-a-half-long workshops held by a dietitian (M.M.) were conducted individually with the participation of girls and their parents, the aim being to provide knowledge about the relationship between the use of an anti-inflammatory diet and health consequences (clinical, metabolic, hormonal, and immunological). Patients received valuable educational materials describing ingredients with anti-inflammatory properties and high antioxidant potential and the possibilities of their use in the daily menu. To unify the rules of intervention (to facilitate intervention compliance) in both study groups, general recommendations regarding the composition of the diet were prepared, taking into account aspects like higher content of (1) vegetable protein from, among others, nuts, grains, seeds, legumes and (2) fibres from plants legumes, cruciferous vegetables, colourful vegetables, fruits, and wholegrain cereals. In addition, based on the principles of the diet, a set of easy-to-follow recipes was prepared for them. These materials were designed based on previous publications^22,26–30^. Throughout the intervention period, at 1–2-week intervals, the dietitian contacted the study participants individually and/or their parents by e-mail and/or telephone to remind them of the rules of the intervention and to provide more detailed and "tailor-made" information recommendations (tailored-made personalized dietary intervention) on improving eating habits according to the principles of AIDiet.

General recommendations about diet composition and AIDiet principles were: vegetables: ≥ 2 portions/day, including ≥ 1 portion raw or in the form of a salad (1 portion: 200 g); fruit, including natural fruit juices: ≥ 3 units per day; legumes: ≥ 3 servings per week (1 serving: 150 g); fish/shellfish: ≥ 3 servings per week (1 serving: 100–150 g fish or 200 g shellfish); nuts, including peanuts: ≥ 1 serving per day (1 serving: 30 g), ≥ 3 units, bread and cereals: 1–2 servings with each meal (1 serving: 25 g bread, 50 g-60 g cooked pasta), eggs: 2–4 servings per week (1 serving: 1 egg), poultry: 2 servings per week (1 serving: 60 g meat), dairy products: 2 servings per day (1 serving: 1 glass of milk or yogurt), red meat: < 2 servings per week (1 serving: 60 g of meat), sweets < 2 servings per week and olive oil: ≥ 4 tablespoons/day (1 tablespoon: 10 g). Instead of wine (typical for MDs), beverages rich in anti-inflammatory polyphenols were recommended, such as green tea, ≥ 1 cup per day^22^, whose benefits were already proven in PCOS^31^. Participants were also trained to consume spices and herbs with high antioxidant and anti-inflammatory potential. Anti-inflammatory spices such as cinnamon, ginger, turmeric, black cumin, and anti-inflammatory herbs: chives, garlic, onion, cloves, rosemary, black pepper, chilies, oregano, thyme, lemongrass were recommended based on Ramirez et al.^29^.

Adherence to dietary recommendations and intervention rules was checked twice, i.e., before the start (T1) and after 12 weeks of its study (T2) using the KIDMED Questionnaire^27,32^. In addition, patients were asked to write a 3-day food diary before and after the intervention to assess their diet regarding energy and macronutrient content^33^.

### Medical, biochemical, and anthropometric assessment

Before the study, during a medical consultation, a gynaecologist, specialised in diseases of the developmental age, assessed all symptoms of hormonal disorders based on anamnesis, physical examination, blood tests, and transabdominal ultrasound (Fig. 1). Anthropometric measurements were performed, including height and weight, and the body mass index (BMI) was calculated. Overweight and obesity were diagnosed and classified based on BMI. Anthropometrical and medical assessment procedures are detailed in^8^.

Blood collection (2 × 7.5 ml) in an appropriate tube was performed after overnight fasting during the early follicular phase (days 3–5) (T1) (Fig. 1). After collection, tubes were spined at 4000 G using a standard room-temperature centrifuge for 10 min. Serum was recovered with a transfer pipette, and placed 0.5 ml of serum into each cryovial tube. Each aliquot was clearly labelled with the appropriate study code and date of collection. The samples were analysed right after preparation. Biochemical parameters, including glucose, insulin, and dehydroepiandrosterone sulphate DHEA-S, were analysed by electrochemiluminescent immunoassay (ECLIA) (Elecsys, Roche Diagnostics GmbH, Mannheim, Germany) at the central laboratory of the Department of Gynaecology and Obstetrics, Poznań University of Medical Sciences. Based on fasting insulin and glucose concentrations, the homeostasis model assessment: insulin resistance and beta-cell function (HOMA-IR index) score was calculated^8^. TNF-α, CRP, IL-1, IL-6, TAC, MDA, and androstenedione concentrations were assayed at the Department of Medical Chemistry and Laboratory Medicine (Poznań University of Medical Sciences). All analytes were assayed in duplicate using commercial ELISA kits. The following assays: IL-1, IL-6, TNF-α, MDA were performed using the commercial ELISA test from SunRed, China. CRP was performed using a commercial ELISA test from DRG, Germany. TAC was performed using a colorimetric (photometric) microplate assay from Omnidiagnostica Forschungs GmbH, Austria, and the results are expressed as mmol/L Trolox equivalent. All the tests were read on a microplate reader TECAN (Switzerland) with software Magellan (Switzerland).

### Dietary assessments

The KIDMED Questionnaire was used to assess the quality of the diet. In addition, the supply of energy and macronutrients was also assessed using the 3-day food record method.

### KIDMED questionnaire

The KIDMED questionnaire (App. A), assessing diet quality in relation to MD dietary patterns, has been validated in studies in many countries as an option for a rapid dietary screening tool in a clinical setting^26,31^. Prior to this study, the questionnaire was translated into Polish. The initial translation from the original language (English) into the target language (Polish) was done by two independent translators^34,35^. Then two independent translators performed back translation^34,36^ to reveal possible translation discrepancies, which were eliminated after consultation with the team.

The KIDMED questionnaire is used to assess the quality of the diet and adherence to the principles of MD (adherence to the Mediterranean Diet). Diet quality is evaluated according to the KIDMED scale (KIDMED index).

The KIDMED index ranges from 0 to 12 points and was prepared based on 16 questions of the KIDMED questionnaire (App. A). Questions with negative connotations concerning MD are assigned a value of − 1, and those with a positive aspect + 1^27^.

According to the KIDMED Index:

- a score of ≥ 8 points is are rated as "high”, indicating optimal diet quality.
- a score of 4–7 points is rated as "medium”, which means the quality of the diet needs improvement.
- a score of ≤ 3 points is rated as "poor”, meaning a very low-quality diet.

### 3-day food diary (three-day food record)

The assessment of the supply of macronutrients and the energy value of the diet was carried out using the method of current recording using a 3-day food diary. This method consists in recording all foodstuffs, meals and beverages consumed, giving home measurements and weights (using the "Album of photos of products and dishes"^37^. The girls were instructed to keep records twice, i.e., at the beginning and end of the research, a detailed three-day food record for two consecutive weekdays and one weekend day. The collected data was analysed by a dietitian using the Aliant Cambridge Diagnostics program. In each case, the daily food intake was compared with the current daily nutritional and energy needs^38^.

### Ethics and dissemination

This study was conducted in accordance with the Declaration of Helsinki and was approved by the Bioethics Committee of the Poznan University of Medical Sciences (consent no. 553/18, ad. 161/20, ad. 416/22). All study participants and their parents were informed about the purpose and method of conducting the study and signed informed consent to participate.

### Sample size calculation

The sample size calculation showed that the necessary size in the study is nine people, assuming equal groups. This estimate was based on the following assumptions: error 5%, power 90%, the mean difference of 3.00 units (points) in the KIDMED score after the nutritional intervention, using similar criteria as used in previous studies, estimate^39^. The proposed size was increased to 20 patients in each group, assuming a drop-out rate of 55%.

### Statistical analysis

The analysis was based on a list of two dependent groups (change over time) separately for overweight and obese women (Ov/Ob.) and separately for non-overweight women and groups (N). When not applied to the application, quantitative data numbers use the normal distribution of the paired t-test and the Wilcoxon matched pairs test. The normality of data distribution was assessed with the Kolmogorov–Smirnov test. Percentage and numerical changes in MD compliance were tested using the Bowker test. A significance level of 0.05 was adopted. Register calculations in the PQStat v1.8.4 program.

### Scheme of the study

The scheme of the study is shown in Fig. 1. Forty girls were subjected to the intervention and were assigned to two groups Ov/Ob (n = 20) or N (n = 20). 32 completed the study, including 19 girls from group N (mean age [years] 15 ± 2) and 13 girls from the Ov/Ob group (average age [years] 16 ± 1). We have excluded from the analysis one girl from the N group because of underweight. Three girls from the Ov /Ob group did not report for testing after three months without giving a reason. Two young women resigned due to family reasons, and the other two girls because of medical problems.

### Patient and public involvement

Patients and/or public were not involved in the design, conduct, reporting or dissemination plans of this research.

### Ethics approval

Examinations were conducted according to the Declaration of Helsinki and accepted by the Bioethics Committee at Medical University in Poznan. They were registered under the number: 553/18 of 14.06.2018 with appendix number 1 of 13.02.2020.

## Results

### Dietary assessment

The assessment of diet quality according to the KIDMED index (before and after the intervention) for N and Ov/Ob groups is presented in Table 1. A statistically significant increase in the KIDMED score was observed in both groups. The median baseline score was 2 in both study groups, while at the end of the intervention, the median scores were 11 in the N group and 9 in the Ov/Ob group. The means and standard deviations (SD) of the scores at the beginning of the study in the N group were 2.11 ± 2.47 and in the Ov/Ob group 1.62 ± 2.69. However, after the intervention, the KIDMED scores in both study groups improved significantly (p < 0.0001) and amounted to 9.95 ± 1.58 vs. 9.92 ± 1.85. An increase in the score after the intervention concerning the period before the intervention was observed in both study groups proves a significant improvement in the quality of the diet and, at the same time, the effectiveness of the intervention (Table 1).

**Table 1:** Quality of diet assessment in KIDMED index score in the groups N and Ov/Ob, before and after intervention.

Measures	(Pre) MD KIDMED score	(Post) MD KIDMED score	p-value
**N (n = 19)**			
Mean ± SD	2.11 ± 2.47	9.95 ± 1.58	** < 0.0001**
**Ov/Ob (n = 13)**			
Mean ± SD	1.62 ± 2.69	9.92 ± 1.85	** < 0.0001**

Data described based on mean and standard deviation, p-value based on paired t-test.

Pre-before the intervention; Post-after the intervention.

Table 2 presents the percentage and/or size changes in girls from both study groups to improve diet quality. Comparing the p-value for the Bowker test of internal symmetry, it was found that the most significant increase in the percentage of subjects in the N and Ov/Ob groups, respectively, was observed in the change from "poor" to "high" (63.2% vs. 61.5%). Slightly less, but also significantly, the percentage of respondents from the "medium" to "high" range increased (31.6% vs. 30.8%), which means that the intervention turned out to be effective, all the more so considering that only a few percent of patients in the N and Ov/Ob group, respectively, improved their knowledge only to the assessment of "medium" (5.3% vs. 7.7) (Table 2).

**Table 2:** Percentage and number of changes in compliance with the MD in the N and Ov/Ob groups.

N (n = 19)	(Post) Adherence to the MD		
(Pre) Adherence to the MD	Poor	Medium	High
Poor	0 (0%)	1 (5.26%)	12 (63.16%)
Medium	0 (0%)	0 (0%)	6 (31.58%)
High	0 (0%)	0 (0%)	0 (0%)
p-value	**0.0026**		

Ov/Ob (n = 13)	(Post) Adherence to the MD		
(Pre) Adherence to the MD	Poor	Medium	High
Poor	0 (0%)	1 (7.69%)	8 (61.54%)
Medium	0 (0%)	0 (0%)	4 (30.77%)
High	0 (0%)	0 (0%)	0 (0%)
p-value	**0.0389**		

p-value based on Bowker test; MD—Mediterranean Diet.

Pre-before the intervention; Post-after the intervention.

Based on the analysis of the patient's diet, it was assessed that the energy value of the diet and the supply of carbohydrates and sugars, animal protein, cholesterol, and total fats, saturated (SFA) and monounsaturated (MUFA) slightly (but not statistically significant) decreased and vegetable protein, polyunsaturated fats (PUFA) and fibre increased in both groups. However, only in the Ov/Ob group, the increase in fibre supply was significant (Table 3).

**Table 3:** Changes in energy and macronutrients intake in the N and Ov/Ob groups.

Variables	N (n = 19)			Ob/Ov (n = 13)		
	Pre	Post	p-value	Pre	Post	p-value
Energy (kcal)	1732.24 ± 716.27	1646.29 ± 427.91	0.6627	1613.16 ± 395.22	1487.77 ± 455.9	0.1190
Protein (g)	74.88 ± 35.79	68.61 ± 20.25	0.5297	69.59 ± 19.7	65.84 ± 20.08	0.5046
Fat (g)	65.39 ± 28.29	63.31 ± 23.84	0.7745	60.47 ± 23.17	56.92 ± 22.02	0.3656
Carbohydrates (g)	219.29 ± 104.71	211.05 ± 62.05	0.7720	205.26 ± 47.26	188.88 ± 56.61	0.1598
Fiber (g)	16.92 ± 12.91	21.12 ± 8.62	0.2355	15.99 ± 7.33	21.11 ± 10.45	**0.0300**
Plant protein (g)	21.58 ± 10.98	24.51 ± 8.35	0.3175	19.42 ± 6.62	22.06 ± 9.05	0.0632
Animal protein (g)	45.93 ± 20.17	39.57 ± 17.2	0.2809	42.1 ± 13.32	39.66 ± 15.8	0.6195
Sugar (g)	42.29 ± 33.97	39.18 ± 19.15	0.7389	48.5 ± 29.6	34.57 ± 20.12	0.0840
SFA (g)	25.67 ± 10.93	25.08 ± 11.52	0.8241	22.26 ± 9.86	21.74 ± 10.25	0.8135
MUFA (g)	23.66 ± 11.44	22.52 ± 10.09	0.7091	21.89 ± 9.3	20.95 ± 7.73	0.5198
PUFA (g)	8.64 ± 4.56	9.45 ± 4.16	0.5873	8.28 ± 3.36	8.36 ± 2.47	0.9117
Cholesterol (mg)	281.13 ± 192.82	244.64 ± 104.57	0.3805	233.05 ± 96.1	229.9 ± 91.28	0.9291

Data described based on Mean ± SD , p-value based on paired t-test; SFA-saturated fatty acids; MUFA-monosaturated fatty acids; PUFA-polyunsaturated fatty acids.

Pre-before the intervention; Post-after the intervention.

### Evaluation of biochemical and anthropometric parameters

The results of our research, presented in Table 4, indicate that as a result of the AIDiet intervention in the N group, a substantial and beneficial decrease in the concentration of inflammatory markers was obtained despite the lack of a significant change in body weight and BMI: IL-1 (p = 0.0001), IL-6 (p = 0.0000), TNF-α (p = 0.0001) and androstenedione levels (p = 0.001). However, in the Ov/Ob group, apart from a significant decrease in the concentrations of IL-1 (0.007), IL-6 (p = 0.008), TNF-α (p = 0.01) and androstenedione (0.0003), we also obtained a significant increase in DHEA-S (0.0172), TAC (p = 0.004) and an apparent decrease in fasting insulin (p = 0.01) and HOMA-IR (p = 0.02). At the same time, the implemented qualitative changes in the diet also significantly impacted the reduction of body weight and BMI, but only in the Ov/Ob group (p = 0.04) (Table 4).

**Table 4:** Changes in anthropometric, hormonal and immuno-metabolic markers in the in the N and Ov/Ob groups.

Variables	N (n = 19)			Ob/Ov (n = 16)		
	Pre	Post	p-value	Pre	Post	p-value
Androstenedione (ng/ml)	4.13 ± 2.89	2.02 ± 0.79	**0.0011**	6.64 ± 3.49	2.12 ± 0.67	**0.0003**
DHEA-S	6.88 ± 2.91	9.54 ± 5.51	0.0504	8.31 ± 3.17	13.25 ± 6.75	**0.0172**
IL-1 (pg/ml)	25.69 ± 9.36	13.49 ± 9.94	**0.0001**	36.32 ± 28.14	17.67 ± 14.53	**0.0066**
IL-6 (ng/l)	29.41 ± 12	10.95 ± 5.97	**0.0000**	45.33 ± 42.11	14.71 ± 9.24	**0.0083**
TNF-α (ng/l)	82.8 ± 37.09	42.48 ± 28.45	**0.0001**	129.38 ± 128.24	49.19 ± 41.04	**0.0114**
CRP (mg/l)	0.86 [0.36; 1.36]	0.47 [0.34; 1.22]	0.9679	1.13 ± 1.27	1.34 ± 1.43	0.2350
TAC (mmol/l)	1.09 ± 0.21	1.43 ± 1.01	0.1618	1.08 ± 0.18	1.81 ± 0.78	**0.0044**
MDA (nmol/ml)	7.71 ± 3.43	8.59 ± 3.53	0.1981	12.63 ± 12.83	10.01 ± 4.6	0.3354
Body mass (kg)	58.11 ± 6.84	57.94 ± 6.55	0.5335	83.82 ± 11.59	80.76 ± 10.65	**0.0406**
BMI (kg/m^2^)	20.86 ± 2.25	20.8 ± 2.09	0.5067	30.14 ± 4.11	29.06 ± 4.02	**0.0406**
Fasting glucose (mg/dL)	87.97 ± 5.6	85.86 ± 6.76	0.2111	92.98 ± 6.56	93.19 ± 5.42	0.9170
Fasting insulin (µU/ml)	14.02 ± 6.91	11.95 ± 4.5	0.1260	22.97 ± 8.46	15.83 ± 4.17	**0.0118**
Homa-IR	3.1 ± 1.69	2.54 ± 1.01	0.0839	5.33 ± 2.15	3.66 ± 1.05	**0.0213**

Data described based on Mean ± SD or Median [Q1; Q3], p-value based on paired t-test or Wilcoxon test; DHEA-S-dehydropiandrostenedione; IL-1-interleukin 1, IL-6-interleukin 2, TNF-α—Tumor Necrosis Factor; CRP-C-reactive protein; TAC-total antioxidant capacity; MDA- malondialdehyde; BMI-body mass index; HOMA-IR—homeostatic model assessment of insulin resistance.

Pre-before the intervention; Post-after the intervention.

## Discussion

Our research showed that the AIDiet dietary intervention, based on the principles of MD, was effective in improving the quality of the diet of girls with PCOS, both slim and overweight or obese. In turn, the improvement in the quality of the diet resulted in a favourable change in the concentration of tested biochemical markers. To our knowledge, this is the first trial based on the assessment of diet quality using the KIDMED scale conducted in girls with PCOS and the first study that has proven that intervention with the AIDiet is associated with a beneficial change in immuno-metabolic and hormonal status.

The MD diet, on which we based the AIDiet intervention, is a diet with high anti-oxidant and anti-inflammatory potential. MD is considered one of the best ways of eating and eating patterns in the context of diet therapy in women with PCOS^41^. Its high anti-inflammatory and antioxidant potential is associated with the high consumption of olive oil rich in polyphenols and oleic acid (which protects lipoproteins and cell membranes against oxidative damage)^42,43^. Long-term consumption of this type of fat may have both a preventive and therapeutic effect on slowing down inflammation in the body (LGI) and, at the same time, a beneficial effect on insulin resistance occurring in PCOS^44^. PUFAs are also anti-inflammatory, present primarily in fatty sea fish species typical for MD but also in walnuts. It has been shown that PUFA taken as supplementation improves insulin resistance and serum lipid concentration and increases adiponectin concentration^41,45^. It has been proven that regular consumption of MD is associated with positive changes in the concentration of markers of inflammation, such as CRP, IL-6, TNF-α as well as clinical and metabolic parameters, including body weight, BMI, WC, blood glucose and insulin^24^.

The 12-week AIDiet intervention implemented in slim, overweight and obese patients resulted in a significant increase in the KIDMED score, proving a substantial improvement in diet quality and in the intervention's effectiveness. The increase in the rate of AID diet compliance was associated with an increase in the supply of plant protein, PUFAs, and fibre. On the other hand, a decrease in the supply of simple sugars, animal protein, and total fats, including saturated fats and cholesterol.

Our results are consistent with those reported by other researchers who conducted an MD-based intervention in children and adolescents to improve diet quality on the KIDMED scale. In studies^39^, the intervention resulted in an increase in the consumption of high-fibre fruit and vegetables and wholegrain cereal products. In addition, nuts are a source of vegetable protein. At the same time, a decrease in the consumption of sweets, meat and sausages, and eggs was observed, and as a result, a reduction in the supply of simple sugars and fats, including SFA^39^.

The dietary intervention we conducted with the participation of teenage PCOS patients was based on our previous research findings^4,8,12,40^. We observed negative but, at the same time, favourable correlations between the concentration of IL-6 and TNF-α, markers of inflammation, and MDA—oxidative stress marker, and the supply of proteins, including plant proteins, carbohydrates, and fibre in overweight and obese PCOS teenagers. Moreover, we showed a positive correlation between dietary cholesterol and CRP, a marker of inflammation, as well as a positive correlation between the concentration of TAC, which is a marker of the body's total antioxidant potential, and the supply of plant protein in lean patients. In none of the groups were we able to show a correlation between the markers being tested and the content of fatty acids in the diet, both SFA, MUFA, and PUFA^8^.

Our previous studies were observational, so the next step was to attempt to confirm these reports in intervention study conducted among girls with PCOS. In our current research we showed that AIDiet intervention resulted in a beneficial and significant decrease in the concentration of the tested cytokines IL-1, IL-6 and TNF-α and androstenedione, along with a significant increase in the quality of the diet, in the group of slim girls (N), despite the lack of a substantial change in body weight and BMI. However, in the overweight and obese group, in addition to a significant improvement in the concentrations mentioned above of the inflammatory markers and androstenedione, we also observed a favorable increase in the concentration of TAC. It is worth noting that an increase in the TAC index means an increase in the concentration of protein and low-molecular antioxidants and, thus, an increase in the body's antioxidant defences. These changes were more obvious in overweight or obese girls because they lost weight, and as is known, inflammation, insulin resistance, and oxidative stress are related to obesity/overweight^8^.

However, in our current research, it was essential to check if intervention with an anti-inflammatory diet, which includes the Mediterranean diet, without introducing changes in body weight (which changes are usually not necessary in slim girls with PCOS) can also lead to achieving beneficial changes in parameters, which we examined, important from the point of view of PCOS.

This is particularly important and it should be emphasized that PCOS also occurs in slim girls, who, however, exhibit common features and metabolic dysfunctions typical of those who are overweight and obese. In these cases, the implementation of reduction diets (one could add "and weight reduction") is unnecessary^16^.

Besides, it should be noted, that, as reported by other authors, certain ingredients with antioxidant activity can reverse inflammation caused by free oxygen radicals (ROS, reactive oxygen species) and OS^8,46–53^. However, we did not expect that, as a result of the intervention, we would obtain an unfavourable increase in the MDA being an oxidative stress index (observed only in the N group) and a slight increase in the concentration of CRP (in the Ov/Ob group). However, in both cases, these changes were statistically insignificant.

As a result of the AIDiet intervention conducted in this study, we also observed a beneficial and significant decrease in the HOMA-IR, insulin resistance index and fasting insulin. This indicates that as a result of the use of an AIDiet in girls with PCOS, metabolic benefits related to improving insulin sensitivity and reducing insulin resistance typical of PCOS can be expected. It is important that these changes can be achieved in slim girls without the need to reduce body weight, which is not justified. It is worth emphasizing that a decrease in fasting insulin concentration and HOMA-IR index was also observed in overweight and obese girls. In this group, it was probably also associated with a significant decrease in body weight and BMI. A number of similar metabolic benefits and the effectiveness of MD-based diet therapy in relation to risk factors and components of the metabolic syndrome were reported by Papadaki A in the Meta-Analysis of Controlled Trials^54^.

It should be emphasized that the intervention conducted in patients with PCOS also resulted in a beneficial and significant change in the concentration of androstenedione (in both study groups). Nevertheless, the results obtained indicate the legitimacy of continuing research related to the influence of diet on the concentration of all androgens. This is important because our previous research showed certain correlations between macronutrients in the diet and the tested androgens in the PCOS and/or control group. In the girls studied, we revealed a positive correlation between the serum concentration of androstenedione and the supply of total fats and MUFA, as well as a negative correlation between the above-mentioned androgen, and the supply of total protein, vegetable protein, and fibre^40^. Intervention studies by other authors showed a beneficial effect of dietary interventions on insulin resistance in PCOS patients and the normalization of hyperandrogenemia^55^. By inducing compensatory hyperinsulinemia, insulin resistance induces several pathways, ultimately leading to subsequent excess of circulating androgens^40,56^. Moreover, a beneficial effect of a 3-month high-protein diet with a low glycaemic load in women with PCOS and excessive body weight on the concentration of CRP, testosterone, and insulin, as well as the HOMA-IR value, was observed^59^. Products with a low load and glycaemic index are usually high in fibre. Previous studies on the relationship between dietary fibre intake and androgen levels and metabolic indices in adult women with PCOS showed that fibre intake was inversely associated with insulin resistance, fasting insulin, glucose, testosterone, and DHEA-S^60^. Unfortunately, in contrast to these findings, our previous studies did not show any significant correlations between fibre intake and androgen levels in girls with PCOS. However, the entire study cohort showed a negative correlation between fibre intake and testosterone and androstenedione concentrations^40^.

The improvement in diet quality in girls with PCOS achieved due to the 12-week AIDiet intervention is significant for teenagers with PCOS because, as we showed previously, their diet differs unfavourably from the control group. These differences relate to the lower supply of fibre and the higher supply of fats, cholesterol, and simple sugars^12^, which are pro-inflammatory ingredients. It is worth mentioning that pro-inflammatory dietary components may promote the occurrence of oxidative stress (OS) and inflammation in the body (LGI)^46,61,63–65^ and affect the concentration of markers of inflammation such as CRP^8,64,66^. Excessive consumption of simple sugars is pro-inflammatory, resulting in elevated CRP levels^67^ and exacerbating insulin resistance, hyperandrogenism, and inflammation in PCOS patients^44^. In addition, certain dietary components, such as glucose, may enhance ovarian androgen production and thus increase inflammation in PCOS^44,68^. The consumption of simple sugars is associated with the occurrence of OS, which stems from an excessive increase in blood glucose concentration as a result of their consumption^69^.

Summing up, the results of the 12-week AIDiet dietary intervention without energy deficit which we conducted in a group of slim and overweight girls with PCOS confirmed our earlier assumptions that a significant increase in the quality of the diet and the supply of ingredients with anti-inflammatory properties might have a beneficial effect on the metabolic and endocrine health of patients and reverse PCOS-associated low-grade inflammation and antioxidant imbalance leading to OS. However, further research is needed in this area.

### Strength and limitation

Our research has some limitations. First, we are aware of the small sample size. The sample size was designed to have sufficient power to assess the primary outcome, the KIDMED indicator. For the secondary outcome, the power was sufficient to detect as statistically significant indicators of inflammation, such as TNF-α, IL-1, IL-6. However, where we did not obtain statistically significant differences, the effects we obtained can serve as a basis for determining the necessary and already larger sample sizes in future studies that will be conducted on this topic.

Secondly, in the diet quality assessment studies, we used a questionnaire which, in principle, is not an entirely objective tool. Nevertheless, the KIDMED questionnaire we used has already been validated in studies in many countries as an option for a rapid dietary screening tool in clinical conditions, and it has been approved for paediatric populations^27,31^. In addition, interviews with patients were conducted face-to-face by an experienced dietitian to minimize possible errors related to completing the questionnaire independently.

Moreover, the dietary checklist does not examine all food types with anti-inflammatory potential. Nevertheless, the choice was based on our previous research findings^4,8,12,40^. In the next follow-up trials, the list should be expanded.

Another limitation of our study is that we did not consider the influence of other lifestyle factors, such as participation in organized physical activity. In addition, the study population was highly homogeneous, as it consisted only of Caucasian girls, and therefore it is difficult to interpolate our results to other populations.

Our attention was also drawn to the high dropout rate in the Ov/Ob group (35%). This suggests that overweight and obese patients and their parents require a particular form of motivation to implement changes in nutrition effectively.

However, it should be emphasized that despite the limitations mentioned above, the girls did not report any adverse events or side effects related to the dietary intervention, which indicates the validity and safety of the diet. Moreover, this is the first study in which a dietary intervention was proposed in girls with PCOS, both slim and overweight and obese, based on the use of a diet with high antioxidant and anti-inflammatory potential (AIDiet) and, at the same time, without the implementation of an energy deficit. In addition, the first study confirmed that the use of AIDiet for 12 weeks is associated with beneficial changes in immuno-metabolic health and hormonal status in adolescent PCOS patients.

## Conclusions

Our research is the first trial based on the assessment of diet quality using the KIDMED scale in girls with PCOS and the first to prove that the improvement in diet quality concerning the KIDMED scale results in a beneficial change in metabolic and hormonal and immunological parameters in this age group of PCOS patients. However, this effect was observed in slim girls without a weight reduction.

Besides, the results show that an AIDiet, based on the assumptions of MD, may play a therapeutic role in teenage PCOS patients, contributing to the reduction in LGI, OS, and insulin resistance, as well as reducing the concentration of androgens.

Further research should contribute to determining whether the use of an AIDiet can achieve health effects related to metabolic and hormonal health in adolescent PCOS patients comparable to pharmacological therapy.

### Supplementary Information

Supplementary Information.

## Data Availability

The datasets generated and/or analysed during the current study are not publicly available due to data were obtained without consent for sharing, but are available from the corresponding author on reasonable request.

## References

[1] The Rotterdam ESHRE/ASRM-Sponsored PCOS consensus workshop group (2004). Revised 2003 consensus on diagnostic criteria and long-term health risks related to polycystic ovary syndrome (PCOS). Hum. Reprod..

[2] González F (2012). Inflammation in polycystic ovary syndrome: Underpinning of insulin resistance and ovarian dysfunction. Steroids.

[3] Azziz R (2006). Androgen Excess Society. Positions statement: Criteria for defining polycystic ovary syndrome as a predominantly hyperandrogenic syndrome: An Androgen Excess Society guideline. J. Clin. Endocrinol. Metab..

[4] Mizgier M (2020). Risk factors of overweight and obesity related to diet and disordered eating attitudes in adolescent girls with clinical features of polycystic ovary syndrome. J. Clin. Med..

[5] Zuo, T. et al. Roles of oxidative stress in polycystic ovary syndrome and cancers. *Oxid. Med. Cell. Longev*. 2016, **14** 10.1155/2016/8589318 PMC4684888 26770659

[6] Agarval A (2013). Studies on women’s health, oxidative stress in applied basic research and clinical practice; Chapter 10: Oxidative stress impact on the fertility of women with polycystic ovary syndrome.

[7] Zhai Y, Pang Y (2022). Systemic and ovarian inflammation in women with polycystic ovary syndrome. J. Reprod. Immunol..

[8] Mizgier M (2021). Relation between inflammation, oxidative stress, and macronutrient intakes in normal and excessive body weight adolescent girls with clinical features of polycystic ovary syndrome. Nutrients..

[9] Repaci A, Gambineri A, Pasquali R (2011). The role of low-grade inflammation in the polycystic ovary syndrome. Mol. Cell. Endocrinol..

[10] Zangeneh FZ, Naghizadeh MM, Masoumi M (2017). Polycystic ovary syndrome and circulating inflammatory markers. Int. J. Reprod. Bio. Med..

[11] Gao L, Gu Y, Yin X (2016). High serum tumor necrosis factor-alpha levels in women with polycystic ovary syndrome: A meta-analysis. PLoS One..

[12] Mizgier M (2021). Dietary and physical activity habits in adolescent girls with polycystic ovary syndrome (PCOS)-HAstudy. J. Clin. Med..

[13] Teede HJ (2018). International PCOS Network. Recommendations from the international evidence-based guideline for the assessment and management of polycystic ovary syndrome. Hum. Reprod..

[14] Moran LJ, Pasquali R, Teede HJ, Hoeger KM, Norman RJ (2009). Treatment of obesity in polycystic ovary syndrome: A position statement of the Androgen Excess and Polycystic Ovary Syndrome Society. Fertil. Steril..

[15] Legro RS (2013). Diagnosis and treatment of polycystic ovary syndrome: An Endocrine Society clinical practice guideline. J. Clin. Endocrinol. Metab..

[16] Satyaraddi A (2019). Body composition, metabolic characteristics, and insulin resistance in obese and nonobese women with polycystic ovary syndrome. J. Hum. Reprod. Sci..

[17] Barrea L (2021). PCOS and nutritional approaches: Differences between lean and obese phenotype. Metabol. Open..

[18] Sureda A (2018). Adherence to the mediterranean diet and inflammatory markers. Nutrients..

[19] Mayr HL (2018). Mediterranean-type diets and inflammatory markers in patients with coronary heart disease: A systematic review and meta-analysis. Nutr. Res..

[20] Lahoz C (2018). Relationship of the adherence to a mediterranean diet and its main components with CRP levels in the Spanish population. Nutrients..

[21] Casas R, Sacanella E, Estruch R (2014). The immune protective effect of the Mediterranean diet against chronic low-grade inflammatory diseases. Endocr. Metab. Immune. Disord. Drug. Targets..

[22] Zuniga KE (2019). Dietary intervention among breast cancer survivors increased adherence to a Mediterranean-style, anti-inflammatory dietary pattern: The Rx for Better Breast Health Randomized Controlled Trial. Breast. Cancer. Res. Treat..

[23] Grosso G (2022). On behalf of the obesity programs of nutrition, education, research and assessment (OPERA) group. Anti-inflammatory nutrients and obesity-associated metabolic-inflammation: state of the art and future direction. Nutrients..

[24] Papadaki A, Nolen-Doerr E, Mantzoros CS (2020). The effect of the mediterranean diet on metabolic health: A systematic review and meta-analysis of controlled trials in adults. Nutrients..

[25] World Health Organization Growth Reference 5–19. Accessed on 03 February 2024; BMI-for-Age for Girls. 2007. https://www.who.int/tools/growth-reference-data-for-5to19-years/indicators/bmi-for-age

[26] Serra-Majem L, Ribas L, García A, Pérez-Rodrigo C, Aranceta J (2003). Nutrient adequacy and mediterranean diet in Spanish school children and adolescents. Eur. J. Clin. Nutr..

[27] Castro-Quezada I, Román-Viñas B, Serra-Majem L (2014). The Mediterranean diet and nutritional adequacy: A review. Nutrients..

[28] Martínez-González MÁ, Hershey MS, Zazpe I, Trichopoulou A (2017). Transferability of the mediterranean diet to non-mediterranean countries what is and what is not the mediterranean diet. Nutrients.

[29] Ramirez AG (2017). An anti-inflammatory dietary intervention to reduce breast cancer recurrence risk: Study design and baseline data. Contemporary. Clinical. Trials..

[30] Bach-Faig A (2011). Mediterranean Diet Foundation Expert Group. Mediterranean diet pyramid today. Science and cultural updates. Public. Health. Nutr..

[31] Maleki V (2021). A comprehensive insight into effects of green tea extract in polycystic ovary syndrome: A systematic review. Reprod. Biol. Endocrinol..

[32] Sahingoz SA, Sanlier N (2011). Compliance with mediterranean diet quality index (KIDMED) and nutrition knowledge levels in adolescents. A case study from Turkey. Appetite..

[33] Gronowska-Senger A (2013). Przewodnik Metodyczny Badań Sposobu Żywienia.

[34] Guillemin F, Bombardier C, Beaton D (1993). Cross-cultural adaptation of health-related quality of life measures: Literature review and proposed guidelines. J. Clin. Epidemiol..

[35] Beaton D, Bombardier C, Guillemin F, Ferraz M (2007). Recommendations for the Cross-Cultural Adaptation of the DASH and Quick DASH Outcome Measures.

[36] Tsang S, Royse CF, Terkawi AS (2017). Guidelines for developing, translating, and validating a questionnaire in perioperative and pain medicine. Saudi J. Anaesth..

[37] Szponar L, Wolnicka K, Rychlik E (2000). Album of Photographs of Food Products and Dishes.

[38] Jarosz M (2020). Nutrition standards for the Polish Population.

[39] Ojeda-Rodríguez A (2018). Improved diet quality and nutrient adequacy in children and adolescents with abdominal obesity after a lifestyle intervention. Nutrients..

[40] Mizgier M (2022). Association of macronutrients composition, physical activity and serum androgen concentration in young women with polycystic ovary syndrome. Nutrients..

[41] Barrea L (2019). Adherence to the mediterranean diet, dietary patterns and body composition in women with polycystic ovary syndrome (PCOS). Nutrients..

[42] Parkinson L, Keast R (2014). Oleocanthal, a phenolic derived from virgin olive oil: A review of the beneficial effects on inflammatory disease. Int. J. Mol. Sci..

[43] Berbert AA, Kondo CR, Almendra CL, Matsuo T, Dichi I (2005). Supplementation of fish oil and olive oil in patients with rheumatoid arthritis. Nutrition..

[44] Gonzalez F, Sia CL, Shepard MK, Rote NS, Minium J (2014). The altered mononuclear cell-derived cytokine response to glucose ingestion is not regulated by excess adiposity in polycystic ovary syndrome. J. Clin. Endocrinol. Metab..

[45] Yang K, Zeng L, Bao T, Ge J (2018). Effectiveness of Omega-3 fatty acid for polycystic ovary syndrome: A systematic review and meta-analysis. Reprod. Biol. Endocrinol..

[46] Stringa N (2017). Relation of antioxidant capacity of diet and markers of oxidative status with C-reactive protein and adipocytokines: A prospective study. Metab. Clin. Exp..

[47] Willcox JK, Ash SL, Catignani GL (2004). Antioxidants and prevention of chronic disease. Crit. Rev. Food Sci. Nutr..

[48] Stahl, W., Sies, H. Antioxidant activity of carotenoids. *Mol. Asp. Med*. **2003**, 24 10.1016/s0098-2997(03)00030-x 14585305

[49] Valko M, Rhodes CJ, Moncol J, Izakovic M, Mazur M (2006). Free radicals, metals and antioxidants in oxidative stress-induced cancer. Chem. Biol. Interact..

[50] Friedman A, Moe S (2006). Review of the effects of omega-3 supplementation in dialysis patients. Clin. J. Am. Soc. Nephrol..

[51] Van Beelen VA (2006). Differential induction of electrophile-responsive element-regulated genes byn-3 and n-6 polyunsaturated fatty acids. FEBS Lett..

[52] Calder PC (2006). N-3 polyunsaturated fatty acids, inflammation, and inflammatory diseases. Am. J. Clin. Nutr..

[53] Lakkur S, Judd J, Goodman M (2015). Oxidative stress, inflammation, and markers of cardiovascular health. Atherosclerosis..

[54] Papadaki A, Nolen-Doerr E, Mantzoros CS (2020). The effect of the mediterranean diet on metabolic health: A systematic review and meta-analysis of controlled trials in adults. Nutrients..

[55] Neves LPP (2020). Nutritional and dietary aspects in polycystic ovary syndrome: Insights into the biology of nutritional interventions. Gynecol. Endocrinol..

[56] Connolly A, Leblanc S, Baillargeon JP (2018). Role of lipotoxicity and contribution of the renin-angiotensin system in the development of polycystic ovary syndrome. Int. J. Endocrinol..

[57] Gower BA (2013). Favourable metabolic effects of a eucaloric lower-carbohydrate diet in women with PCOS. Clin. Endocrinol..

[58] Douglas CC, Gower BA, Darnell BE, Ovalle F, Oster RA, Azziz R (2006). Role of diet in the treatment of polycystic ovary syndrome. Fertil. Steril..

[59] Mehrabani HH, Salehpour S, Meyer BJ, Tahbaz F (2012). Beneficial effects of a high-protein, low-glycemic-load hypocaloric diet in overweight and obese women with polycystic ovary syndrome: A randomized controlled intervention study. J. Am. Coll. Nutr..

[60] Cutler DA, Pride SM, Cheung AP (2019). Low intakes of dietary fiber and magnesium are associated with insulin resistance and hyperandrogenism in polycystic ovary syndrome: A cohort study. Food Sci. Nutr..

[61] Ren Z (2018). Association between dietary inflammatory index, c-reactive protein and metabolic syndrome: A cross-sectional study. Nutrients..

[62] Christ A, Lauterbach M, Latz E (2019). Western diet and the immune system: An inflammatory connection. Immunity..

[63] Ma K, Jin X, Zhao Q, Zhang X (2012). Inflammatory mediators involved in the progression of the metabolic syndrome. Diabetes/Metab. Res. Rev..

[64] Shivappa N (2015). Associations between dietary inflammatory index and inflammatory markers in the Asklepios Study. Br. J. Nutr..

[65] Garcia-Arellano A (2015). Dietary inflammatory index and incidence of cardiovascular disease in the PREDIMED study. Nutrients..

[66] Valko M (2007). Free radicals and antioxidants in normal physiological functions and human disease. Int. J. Biochem. Cell Biol..

[67] Levitan EB (2008). Dietary glycemic index, dietary glycemic load, blood lipids, and C-reactive protein. Metabolism..

[68] Gonzalez F (2015). Nutrient-induced inflammation in polycystic ovary syndrome: Role in the development of metabolic aberration and ovarian dysfunction. Semin. Reprod. Med..

[69] Barrea L (2018). Source and amount of carbohydrate in the diet and inflammation in women with polycystic ovary syndrome. Nutr. Res. Rev..

