# Supplementary material for: Effects of AIDiet intervention to improve diet quality, immuno-metabolic health in normal and overweight PCOS girls: a pilot study
Source: Sci Rep. 2024 Feb 12;14:3525. doi: 10.1038/s41598-024-54100-1 (PMC10861446; doi:10.1038/s41598-024-54100-1)
Supplement: Supplementary file 1 — Supplementary Information. [file 41598_2024_54100_MOESM1_ESM.docx]

Appendix A

Supplementary table 1. KIDMED test to assess Mediterranean diet

KIDMED test Scoring

| Takes a fruit or fruit juice every day | +1 |
| --- | --- |
| Has a second fruit every day | +1 |
| Has fresh or cooked vegetables regularly once a day | +1 |
| Has fresh or cooked vegetables more than once a day | +1 |
| Consumes fish regularly (at least 2–3/week) | +1 |
| Goes >1/week to a fast food restaurant (hamburger) | −1 |
| Likes pulses and eats them >1/week | +1 |
| Consumes pasta or rice almost every day (5 or more per week) | +1 |
| Has cereals or grains (bread, etc.) for breakfast | +1 |
| Consumes nuts regularly (at least 2–3/week) | +1 |
| Uses olive oil at home | +1 |
| Skips breakfast | −1 |
| Has a dairy product for breakfast (yoghurt, milk, etc.) | +1 |
| Has commercially baked goods or pastries for breakfast | −1 |
| Takes two yoghurts and/or some cheese (40 g) daily | +1 |
| Takes sweets and candy several times every day | −1 |

KIDMED Index: poor ≤ 3; medium 4-7; high ≥ 8
